# Assessing physical therapist students’ self-efficacy: measurement properties of the Physiotherapist Self-Efficacy (PSE) questionnaire

**DOI:** 10.1186/s12909-017-1094-x

**Published:** 2017-12-12

**Authors:** Wim van Lankveld, Anne Jones, Jaap J. Brunnekreef, Joost P. H. Seeger, J. Bart Staal

**Affiliations:** 1 HAN University of Applied Sciences, Research group Musculoskeletal Rehabilitation, Kapittelweg 33, Nijmegen, The Netherlands; 2 College of Health Care Sciences, James Cook University, Douglas, Australia; 3 Radboud University Medical Centre, Radboud Institute for Health Sciences, IQ healthcare, Nijmegen, the Netherlands

**Keywords:** Physical therapist self-efficacy, Education, Physical therapy students

## Abstract

**Background:**

Apart from skills, and knowledge, self-efficacy is an important factor in the students’ preparation for clinical work. The Physiotherapist Self-Efficacy (PSE) questionnaire was developed to measure physical therapy (TP) students’ self-efficacy in the cardiorespiratory, musculoskeletal, and neurological clinical areas. The aim of this study was to establish the measurement properties of the Dutch PSE questionnaire, and to explore whether self-efficacy beliefs in students are clinical area specific.

**Methods:**

Methodological quality of the PSE was studied using COSMIN guidelines. Item analysis, structural validity, and internal consistency of the PSE were determined in 207 students. Test-retest reliability was established in another sample of 60 students completing the PSE twice. Responsiveness of the scales was determined in 80 students completing the PSE at the start and the end of the second year. Hypothesis testing was used to determine construct validity of the PSE.

**Results:**

Exploratory factor analysis resulted in three meaningful components explaining similar proportions of variance (25%, 21%, and 20%), reflecting the three clinical areas. Internal consistency of each of the three subscales was excellent (Cronbach’s alpha > .90). Intra Class Correlation Coefficient was good (.80). Hypothesis testing confirmed construct validity of the PSE.

**Conclusion:**

The PSE shows excellent measurement properties. The component structure of the PSE suggests that self-efficacy about physiotherapy in PT students is not generic, but specific for a clinical area. As self-efficacy is considered a predictor of performance in clinical settings, enhancing self-efficacy is an explicit goal of educational interventions. Further research is needed to determine if the scale is specific enough to assess the effect of educational interventions on student self-efficacy.

## Background

A requirement of any Health Professional educational program is that it must prepare students to meet the demands of clinical practice. The entry level for physical therapist (PT) requires self-determined, professional and clinical decision making in the face of an ever-increasing body of knowledge [1]. Therefore, self-efficacy, or growing task specific confidence, is considered critical for professional development in health professional students [2–4]. Self-efficacy is defined as a person’s beliefs in their capability to organize and execute courses of action required to attain designed types of performance [5]. In education research self-efficacy refers to the students beliefs about his or her capacities to perform certain tasks [6]. Self-efficacy is recognized as an important factor related to academic performance [7, 8]. In PT, self-efficacy is considered an independent predictor for student performance in clinical settings [9].

A number of studies have addressed self-efficacy in PT. High fidelity simulation of acute care settings has been demonstrated to improve PT students’ self-efficacy specific for acute care clinical practice [9–13]. In one study a short Motivational Interviewing learning module improved the students’ self-efficacy towards physical activity counseling [3]. Self-efficacy beliefs towards functioning as a PT was also improved by engaging senior students as standardized patients [14]. All these studies used self-developed and clinical area specific self-efficacy scales. In line with one of the most important recommendation of Bandura [15], such specific self-efficacy scales are thought to be more predictive for the behavior under study than general self-efficacy scales. Unfortunately, these self-developed scales often rely on face validity, and additional measurement properties have not been reported. The lack of validated outcome measures is a problem for education research in physical therapy [16]. A challenge for education research in PT is to progress beyond single-site studies using validated outcome measures [17, 18]. As self-efficacy towards functioning as a PT is an important outcome measure, there is a need to develop validated and reliable outcome measures to assess self-efficacy in PT. Such measures allow for comparisons between different educational methods and interventions, and might help to progress evidence based PT education.

Only two studies have reported on measurement properties in PT measures of self-efficacy. For students in manual medicine programs, scales were developed to measure communication and clinical skills [19]. The questions in these scales reflect interactions and experiences with patients that students were likely to encounter, and ranged from discussing general health issues to performing basic and focused physical examination procedures. The psychometric analysis of two sub scales, Patient Communication Confidence Scale (PCCS) and the Clinical Skills Confidence Scale (CSCS), was assessed in 269 students. Analyses showed that the scales provided valid and reliable measures of confidence for this sample of persons. Another study described the development of the physiotherapy self-efficacy (PSE) questionnaire to assess self-efficacy in acute care [9]. The scale used in the study of Jones et al. (2012) consisted of 13 statements reflecting key criteria that students would be assessed on whilst on their acute care clinical placement. A panel of five practicing clinicians, with experience both as educators and PT in acute care, were asked to review the items. The panel confirmed the content validity of the scale, in which every item is an effect indicator of self-efficacy. However, only 16 third year undergraduate students completed the final questionnaire, and analysis is limited to item descripives and correlations. In an updated version of the questionnaire, the same 13 questions were asked three times related to three distinct clinical areas of physical therapy: cardiorespiratory, musculoskeletal, and neurological [20]. However, the questionnaire is not available in Dutch and the measurement properties of this extended version have not been studied. Using the extended 39 item PSE to assess self-efficacy beliefs in three distinct clinical areas will help determine whether PT student’s self-efficacy beliefs regarding key criteria of student function are independent of clinical area or clinical area specific. When self-efficacy beliefs are independent of clinical area, self-efficacy beliefs on functioning in one clinical area might be transferred to other clinical areas. This could for example mean that self-efficacy beliefs acquired about the ability to treat a patients with musculoskeletal condition would transfer into self-efficacy beliefs about the ability to treat patients with other conditions. On the other hand, if self-efficacy is specific for one clinical area this transfer of self-efficacy beliefs to other clinical areas is unlikely to happen. Either way, these findings might have important implications for physical therapy training.

Therefore, the general aim of this study is to investigate measurement properties of the extended Physiotherapy Self-Efficacy (PSE) questionnaire in Dutch. In addition, a specific aim of the study is to explore whether physical therapy self-efficacy beliefs on key criteria of functioning assessed with the PSE is independent of clinical area or clinical area specific.

## Methods

Measurement properties of the cross-cultural adapted PSE were assessed in accordance with the **C**onsensus-based **S**tandards for the selection of health Measurement Instruments (COSMIN) [21]. The translated version of the PSE was tested in a number of different student samples to determine validity, reliability, and responsiveness.

### Participants

To determine measurement properties, three distinct convenience samples were used drawn from the present and past PT student population of the HAN University of Applied Sciences in Nijmegen, The Netherlands. Students were invited to participate in the study by mail, and only those student responding by mail to the invitation were asked to complete the questionnaire after giving informed consent. In 2014, the first sample was drawn from students in the third and fourth year of their study (invitational mails were send to 250 students), and from practicing physical therapists recently graduated from the HAN (80 alumni less than 2 year after graduation were invited by mail). Students in this cross-sectional sample were asked by mail to indicate their willingness to participate in a validation study requiring the completion of an online questionnaire. A second sample was drawn from the population of second year students in 2015. These students (*N* = 120) were invited to participate in a longitudinal study into changes in self-efficacy over 1 year. Participants were informed that they had to complete an online questionnaire twice: at the beginning and the end of the study year. The curriculum in the second year includes case studies and practice with simulated patients from all three clinical areas (cardiorespiratory, musculoskeletal, and neurological). Therefore it is expected that self-efficacy beliefs towards these clinical areas will improve in year two. In 2016, a third sample of students was drawn from all first and second year students. Mailed invitations were send to 330 students asking them to participate in a study that would require them to be present at the HAN University of Applied Science for two pre-scheduled appointments 1 week apart. This procedure ensured that all participating students completed the questionnaire twice, with an interval of 1 week. The first 60 students that reacted to the mail were invited for the study. After giving their informed consent, students were enrolled in the study. In the first two samples, participants were directed to a website for completion of the questionnaires. In the third sample, students completed the questionnaire on paper as the web based programme could not handle all students simultaneously.

### Measurements

All three samples completed the PSE as well as reporting gender and age. Sample 1 and 2 were asked to complete an additional questionnaire.

#### Physical therapy self-efficacy (PSE)

The developer of the original instrument (Jones) granted permission for this cross-cultural adaptation into Dutch using Beaton’s method for cross cultural adaptation [22]. The revised PSE measures self-efficacy beliefs in three clinical areas with 39 five point Likert items. The participants were asked to indicate their confidence to perform the described task (1 = very little confidence; 5 = a lot of confidence). This sample of students was asked to complete the questionnaire using an internet tool (Survey Monkey*®, 2014*). After completing the questionnaire, the students were contacted by phone to establish acceptability and suggestions for revisions. The instrument takes on average 10 min to complete.

#### General self-efficacy

The first sample completed the Dutch General Self-efficacy (D-GSES) scale [23]. The D-GSES measures general self-efficacy using 10 statements on a four point Likert scale. The scale is designed to assess optimistic self-beliefs to cope with a variety of difficult demands in life. The items reflect a person’s self-efficacy beliefs not tied to specific behaviors or situations [24]. Higher scores indicate higher levels of self-efficacy.

#### Self-efficacy related to work/study

In the second sample, students completed the PsyCap [25, 26]. The PsyCap measures Psychological Capability related to work/study in four distinct dimensions: self-effectivity, hope, optimism, and resilience. The PsyCap consists of 22 items to be scored on a six point Likert scale (1 = strongly disagree; 6 = strongly agree). For this study, only the six item self-effectivity subscale of the PsyCap was used. Self-effectivity in the PsyCap is defined as an individual’s confidence in their ability to mobilize their motivation, cognitive resources and courses of action to achieve high levels of work related performance [27]. Higher scores reflect higher levels of psychological capability.

### Data analysis

Data from sample one and two were gathered using a web based programme. Once logged in, the students were required by the system to answer every question thus preventing missing items. Data from sample three were completed using printed questionnaires and checked and corrected for missing data on completion by students involved in the project. Descriptive statistics of student samples for ordinal and nominal data are given including proportions. Associations between continuous variables were analysed using Pearson correlation (r).

*Cross cultural adaptation* using the Beaton method is a stepwise apporach [22]. In step one, the original 39 item instrument in English was translated into Dutch by two independent translators not related to the study. In step two, an expert panel discussed the different translations by e-mail until consensus was reached. The expert panel included both translators, the researcher responsible for the project (WvL), and three students participating in the project. In step three, this synthesized version was back translated into English by two different independent translators. Both translators worked independently from each other, but compared their translations and reported on the differences in translation to the expert panel. At step four, an expert committee including all four translators and two researchers discussed the final version of the back-translation by e-mail. In a final step *content validity* of the translated version in Dutch was checked using a sample of 20 undergraduate students.

*Item analysis* was performed on the combined data from samples one and two, calculating Standard Deviation (SD), range of observed scores, and skewness for each of the PSE items. Skewness is a measure of symmetry of frequency distribution, and values between −2 and +2 indicate normal univariate distribution [28].

C*onstruct or structural validity* of the PSE is explored with Exploratory factor analysis (EFA), using principle component analysis as a dimension reduction technique [29]. To this end, sampling adequacy was determined first. The Kaiser-Meyer-Olkin (KMO) was calculated to determine if the variables included in the scale depict a common factor. A KMO value > .8 is considered good, indicating that a principal component analysis is useful in this condition. Next, the Bartlett test of sphericity was conducted. When significant, the test shows that distinct items can be summarized in underlying factors. Finally, principal component analysis using Varimax rotation and maximum likelihood extraction was used as a dimension reduction technique. To determine the number of underlying dimensions, or components, the following strategies were used: the Kaiser criterion (Eigenvalue >1), interpretation of the Scree plot, and cumulative percent of variance extracted [29]. Significance of factor loadings was derived from Stevens [30]. Next, several measures of reliability were computed.

*Internal consistency* of the three subscales of the PSE was determined by calculating Cronbach’s Alpha reflecting the degree of interrelatedness among items. A Cronbach’s alpha >0.75 is considered good [31]. Total scores for the three subscales were calculated by calculating average item scores.

*Test-retest Reliability* was determined in the sample of second year students completing the questionnaire twice with an interval of 1 week. Mean differences between test and re-test item scores were calculated with corresponding 95% confidence interval (CI). When zero lies within the 95% CI this is considered a criterion for absolute agreement. Finally, for each PSE scale Intra Class Correlation (ICC) between both assessments was calculated with corresponding 95% CI, to determine absolute agreement between assessments. The random effects model was used. An ICC above 0.75 is considered good [31]*.* The same data were used to calculate *measurement error.* The value of the Standard Error of Measurement (SEM) was calculated by dividing the SD of the mean differences between two measurements by √2 [32].

*Responsiveness* was determined in sample 2 in which PSE was assessed twice: once at the start of the year and the second time at the end of the year. Change in scale scores over time was tested using student T-test for paired samples. The scale is considered responsive if the observed change is larger than the SEM.

*Criterion validity* of the PSE was determined using *Hypothesis testing*. As there is no gold standard for PT self-efficacy to determine construct or criterion validity, a number of hypotheses were formulated about the PSE. It is expected that self-efficacy is clinical area specific, and that self-efficacy beliefs in the three clinical areas will only be moderately interrelated. Gender or age of the respondents are expected to be unrelated to self-efficacy beliefs. Finally, it is expected that self-efficacy beliefs related to specific clinical areas are only weakly related to general self-efficacy assessed with the D-GSES, and self-efficacy related to work/study assessed with the PsyCap. The strength of correlations is defined as negligible (0.00 to 0.30), low (0.30 to 0.50), moderate (0.50 to 0.70), high (0.70 to 0.90), very high (.90 to 1.00) [33]. The construct validity of the scale is considered good when >75% of the hypothesis can be confirmed [31].The Statistical Package for the Social Sciences (SPSS) version 21 was used for statistical analysis, and a value of *p* < .05 was considered statistical significant.

## Results

The cross cultural adaptation of the PSE was endorsed by the original author. The pilot test and results were discussed by the expert panel and resulted in some minor changes.

### Item analyses

Data from sample one and baseline assessment of sample two were combined for these analyses. A total of 207 students and ex-students completed the 39 item PSE: 116 s year students participated, 39 third year students, 23 fourth year students, and 29 Alumni. The ratio female/male was 64/36. The average age of this sample was 21.3 (SD = 3.4). In Table 1, the wording of the distinct items of the PSE are given. The same 13 items were repeated for the cardiorespiratory, musculoskeletal, and neurological caseload.

**Table 1:** Wording of the 13 items for each PSE dimension. All items are asked in relation to the cardiorespiratory, musculoskeletal, and neurological caseload dimension

Item	Wording of the items.
1	I feel adequately prepared to undertake a….. caseload.
2	I feel that I am able to verbally communicate effectively and appropriately for a …. caseload.
3	I feel that I am able to communicate in writing effectively and appropriately for a …. caseload.
4	I feel that I am able to perform subjective assessments for a …. caseload.
5	I feel that I am able to perform objective assessment for a ….caseload.
6	I feel that I am able to interpret assessment findings appropriate for a …. caseload.
7	I feel that I am able to identify and prioritize patient’s problems for a …. caseload.
8	I feel that I am able to select appropriate short and long term goals for a …. caseload.
9	I feel that I am able to appropriately perform treatments for a …. caseload.
10	I feel that I am able to perform discharge planning for a …. caseload.
11	I feel that I am able to evaluate my treatments for a …. caseload.
12	I feel that I am able to progress interventions appropriately for a ….caseload.
13	I feel that I am able to deal with the range of patient conditions which may be seen with a ….caseload.

Average scores for individual items ranged from 2.86 (I feel that I am able to perform discharge planning for a neurological caseload) to 4.00 (I feel that I am able to verbally communicate effectively and appropriately for a musculoskeletal caseload). Each of the 39 items of the PSE showed univariate normal distribution with values for skewness for all items close to zero. None of the items were excluded for further analysis.

#### Construct validity

The Kaiser-Meyer-Olkin (KMO) test of all 39 items was 0.94, indicating that a principal component analysis was useful in this condition. The Bartlett test of sphericity was significant at 0.000, indicating that distinct items could be summarized in underlying factors [29]. The EFA resulted in four components with an Eigenvalue >1.00 (Eigenvalues respectively 17.0, 4.9, 3.6, and 1.1). The first three components each explained substantial proportions of variance (25%, 21%, and 19% respectively), with the fourth component explaining 3% of the variation. Based on the Scree plot it was estimated that the break occurred at component 3. Therefore, an additional EFA was conducted setting the number of factors at three. The results from the principal component analyses using Varimax rotation with a three factor solution are depicted in Table 2. Statistical significant item loadings are depicted in bold.

**Table 2:** Factor loading of PSE items in a rotated three factor principal component analysis (*N* = 207). Significant loading in bold

		Component		
Dimension		1	2	3
I feel adequately prepared to undertake a **cardiorespiratory** caseload.	1	0.11	0.16	**0.73**
I feel that I am able to verbally communicate effectively and appropriately for a **cardiorespiratory** caseload.	2	0.26	0.26	**0.61**
I feel that I am able to communicate in writing effectively and appropriately for a **cardiorespiratory** caseload.	3	0.24	0.20	**0.58**
I feel that I am able to perform subjective assessments for a **cardiorespiratory** caseload.	4	0.20	0.19	**0.73**
I feel that I am able to perform objective assessment for a **cardiorespiratory** caseload.	5	0.21	0.16	**0.75**
I feel that I am able to interpret assessment findings appropriate for a **cardiorespiratory** caseload.	6	0.15	0.15	**0.77**
I feel that I am able to identify and prioritize patient’s problems for a **cardiorespiratory** caseload.	7	0.28	0.20	**0.65**
I feel that I am able to select appropriate short and long term goals for a **cardiorespiratory** caseload.	8	0.26	0.17	**0.76**
I feel that I am able to appropriately perform treatments for a **cardiorespiratory** caseload.	9	0.21	0.19	**0.78**
I feel that I am able to perform discharge planning for a **cardiorespiratory** caseload.	10	0.23	0.20	**0.70**
I feel that I am able to evaluate my treatments for a **cardiorespiratory** caseload.	11	0.17	0.16	**0.78**
I feel that I am able to progress interventions appropriately for a **cardiorespiratory** caseload.	12	0.27	0.12	**0.79**
I feel that I am able to deal with the range of patient conditions which may be seen with a **cardiorespiratory** caseload.	13	0.31	0.16	**0.65**
I feel adequately prepared to undertake a **musculoskeletal** caseload.	1	0.18	**0.75**	0.16
I feel that I am able to verbally communicate effectively and appropriately for a **musculoskeletal** caseload.	2	0.28	**0.65**	0.31
I feel that I am able to communicate in writing effectively and appropriately for a **c musculoskeletal** caseload.	3	0.25	**0.66**	0.19
I feel that I am able to perform subjective assessments for a **musculoskeletal** caseload.	4	0.08	**0.78**	0.12
I feel that I am able to perform objective assessment for a **musculoskeletal** caseload.	5	0.06	**0.80**	0.15
I feel that I am able to interpret assessment findings appropriate for a **musculoskeletal** caseload.	6	0.14	**0.76**	0.22
I feel that I am able to identify and prioritize patient’s problems for a **musculoskeletal** caseload.	7	0.14	**0.80**	0.06
I feel that I am able to select appropriate short and long term goals for a **musculoskeletal** caseload.	8	0.16	**0.75**	0.19
I feel that I am able to appropriately perform treatments for a **musculoskeletal** caseload.	9	0.07	**0.81**	0.20
I feel that I am able to perform discharge planning for a **musculoskeletal** caseload.	10	0.20	**0.68**	0.16
I feel that I am able to evaluate my treatments for a **musculoskeletal** caseload.	11	0.14	**0.77**	0.20
I feel that I am able to progress interventions appropriately for a **musculoskeletal** caseload.	12	0.13	**0.79**	0.25
I feel that I am able to deal with the range of patient conditions which may be seen with a **musculoskeletal** caseload.	13	0.27	**0.70**	0.16
I feel adequately prepared to undertake a **neurology** caseload.	1	**0.81**	0.21	0.10
I feel that I am able to verbally communicate effectively and appropriately for a **neurology** caseload.	2	**0.76**	0.18	0.25
I feel that I am able to communicate in writing effectively and appropriately for a **neurology** caseload.	3	**0.81**	0.18	0.26
I feel that I am able to perform subjective assessments for a **neurology** caseload.	4	**0.82**	0.16	0.20
I feel that I am able to perform objective assessment for a **neurology** caseload.	5	**0.80**	0.18	0.23
I feel that I am able to interpret assessment findings appropriate for a **neurology** caseload.	6	**0.83**	0.17	0.24
I feel that I am able to identify and prioritize patient’s problems for a **neurology** caseload.	7	**0.83**	0.18	0.20
I feel that I am able to select appropriate short and long term goals for a **neurology** caseload.	8	**0.82**	0.17	0.23
I feel that I am able to appropriately perform treatments for a **neurology** caseload.	9	**0.83**	0.18	0.28
I feel that I am able to perform discharge planning for a **neurology** caseload.	10	**0.78**	0.15	0.26
I feel that I am able to evaluate my treatments for a **neurology** caseload.	11	**0.79**	0.19	0.32
I feel that I am able to progress interventions appropriately for a **neurology** caseload.	12	**0.83**	0.17	0.29
I feel that I am able to deal with the range of patient conditions which may be seen with a neurology caseload.	13	**0.83**	0.09	0.22

All items had statistical significant loading on only one of the three components. All items referring to self-efficacy beliefs about functioning in the neurological clinical area loaded on the first component explaining 25% of the variation. All items referring to self-effectivity beliefs in the musculoskeletal area loaded on the second component (21%). Finally the third component depicts self-efficacy beliefs in functioning in the cardiorespiratory area (20%). Together the three components explained 65.6% of the total variation on all items.

#### Internal consistency

Items with statistical significant loadings on one component were used to compute separate scale scores reflecting self-efficacy related to the cardiorespiratory, musculoskeletal, and neurological clinical areas. Internal consistency expressed in Cronbach’s Alpha’s for the three sub-scales is high (0.94, 0.95, 0.97 for cardiorespiratory, musculoskeletal, and neurological caseload). For each of the subscales average item scores were computed, and these average scores were used in further analysis. Intercorrelation between the subscales ranged from .44 (neurological – musculoskeletal caseload) to 0.57 (neurological – cardiorespiratory caseload).

#### Test retest reliability

Test-retest reliability for the three subscales was computed in the third sample consisting of 60 students (average age = 19 years; 55% females; 75% second year students and 25% first year students). Table 3 shows the average scores on both assessments that were completed within 1 week.

**Table 3:** Test retest difference for each of the three caseload PSE subscales with Intra Class Correlation and Standard Error of Measurement assessed in 60 students with an interval of one week

	1st (SD)	2nd (SD)	Mean difference	95%	ICC	95%	SEM
Cardiorespiratory	3.0 (0.76)	3.1 (0.85)	0.07 (0.52)	−0.2-0.6	0.80	0.70–0.88	0.37
Musculoskeletal	3.4 (0.60)	3.3 (0.58)	0.12 (0.37)	−0.1 − 0.2	0.79	0.67–0.87	0.27
Neurological	2.5 (0.74)	2.5 (0.72)	0.10 (0.47)	-0.2-0.1	0.80	0.68–0.88	0.33

*1st* first assessment, *2nd* second assessment, *ICC* Intra Class Correlation, *SEM* Standard Error of Measurement

For each of the three subscales zero lay between the 95% CI of the mean difference. Absolute agreement assessed using ICC between both assessments of each of the three subscales was > .75, indicating excellent reliability.

#### Responsiveness

The longitudinal data from sample two were used to determine responsiveness. At baseline 116 students completed the questionnaire, and 80 students at the end of that year. Only data from these 80 students were used to determine responsiveness. Table 4 shows average scores at the beginning and at the end of year two, average change between both assessments, and T- statistics for pairwise comparison.

**Table 4:** Change in PSE subscale scores between beginning and the end of year two (*N* = 80)

	Start year 2	End year 2	Change	T
Cardiorespiratory	3.1 (0.68)	3.6 (0.44)	0.53	8.9**
Musculoskeletal	3.7 (0.56)	3.9 (0.36)	0.26	3.2*
Neurological	2.8 (0.79)	3.5 (0.50)	0.73	9.2**

*p < 0.05; ** *p* < 0 .01

Comparing the average student scores at the start of year two with average scores of the same students at the end of year two resulted in statistical significant improvement in self-efficacy for cardiorespiratory, musculoskeletal, and neurological clinical areas. The change in average scores exceeded the SEM for the self-efficacy scales related to cardiorespiratory and neurological clinical areas, but not for the musculoskeletal clinical area.

#### Hypothesis testing

In Table 5 the a priori formulated hypotheses to be tested are given, together with their confirmation (+), or rejection (−). Gender and age were unrelated to PSE scales in any of the samples. In sample 1 all participants completed the D-GSES resulting in an average item score of 3.1 (SD = 0.4) on a four point scale. D-GES average scores showed low positive pearson correlations with PSE clinical area specific self-efficacy (*r* = 0.34, *p* < 0.01; *r* = 0.45, *p* < 0.01; *r* = 0.30 p < 0.01 for cardiorespiratory, musculoskeletal, and neurological clinical areas). Average PscyCap item score in sample 2 was 4.45 (SD = 0.54) on a six point likert scale. Low correlations were found between PSE subscales and job-related self-efficacy assessed using the PsyCap (0.20, *p* < 0.05; 0.19, *p* < 0.05; 0.10, *p* = ns respectively). All correlations between PSE subscales for self-efficacy beliefs in the cardiorespiratory, musculoskeletal, and neurological clinical areas and other indicators of self-efficacy were weak to moderate.

**Table 5:** Construct validity based on a-priori hypothesis (confirmation/rejection = +/−)

1	PT self-efficacy is clinical area specific: PSE items related to one clinical area will load significantly on one dimension in the Principal Component Analysis, and not on the other dimensions.	+
2	The PSE three subscales will have low to moderate intercorrelations (.30 < *r* < .70.	+
3	Gender is unrelated to any of the PSE subscales.	+
4	Age is unrelated to any of the PSE subscales.	+
5	The PSE Cardiovascular subscale score will show low positive correlation (*r* < .50) with general efficacy assessed with D-SES.	+
6	The PSE Cardiovascular subscale score will show low positive correlation (r < .50) with PsyCap subscale Self efficacy.	+
7	The PSE Musculoscelethal subscale score will show low positive correlation (r < .50) with general efficacy assessed with D-GSES.	+
8	The PSE Musculoscelethal subscale score will show low positive correlation (r < .50) with PsyCap subscale Self efficacy.	+
9	The PSE Neurological subscale score will show low positive correlation (r < .50) with general efficacy assessed with D-GSES.	+
10	The PSE Neurological subscale score will show low positive correlation (r < .50) with PsyCap subscale Self-efficacy.	+

More than 75% of the a-priori formulated hypothesis were confirmed.

## Discussion

Self-efficacy is important in preparing PT students for clinical practice [2, 3, 9]. This is the first known study to report measurement properties of a Dutch instrument to measure student self-efficacy for clinical area specific PT functioning in clinical practice. The PSE questionnaire was found to be a valid and reliable instrument to assess PT self-efficacy in key criteria of functioning in the cardiorespiratory, musculoskeletal, and neurological clinical areas. Three resulting subscales assessing self-efficacy towards these three clinical areas had excellent internal consistency, and high test-retest reliability. Responsiveness of the subscales was confirmed on two of the scales. Furthermore, construct validity was confirmed in hypothesis testing.

With regard to the responsiveness of the instrument, the change in self-efficacy towards the musculoskeletal clinical area in the sample of second year students did not exceed SEM. In this study, self-efficacy beliefs related to the musculoskeletal clinical area at the start of year two was higher compared to self-efficacy beliefs related to the cardiorespiratory and musculoskeletal and neurological clinical areas. In hindsight, this was to be expected, as the first year of the educational curriculum at HAN UAS is strongly focused on musculoskeletal conditions, with lesser attention to conditions in the other clinical areas. In year two cardiorespiratory and neurological caseloads are the focus of attention, and therefore, the strong increase in self-efficacy towards these caseloads are to be expected.

An important finding of this study is that PT students’ self-efficacy beliefs in key criteria of functioning are clinical area specific. Earlier studies have described PT self-efficacy scales to measure self-efficacy in specific settings, for instance manual medicine [19], or acute care [9]. The PSE takes a different approach, in that it measures self-efficacy beliefs about key criteria of function in distinct clinical areas. In the clinical phase of their study, students are pre-dominantly confronted with cardiorespiratory, musculoskeletal, and neurological caseloads, hence the decision to focus on these three clinical dimensions. The EFA on the items show that the PSE clearly reflects distinct components in self-efficacy beliefs, with three components related to the cardiorespiratory, musculoskeletal, and neurological clinical areas. This means that a student might have high self-efficacy beliefs on key criteria of functioning in one clinical area, and low self-efficacy beliefs in the same key criteria in the other clinical areas. For instance, a student might have high self-efficacy beliefs about communication skills (PSE items 2 and 3) when confronted with a patient with musculoskeletal conditions, and at the same time have low self-efficacy beliefs in the same communication skills towards patients with a neurological condition. This has important implications for PT education. To improve self-efficacy towards clinical areas, high fidelity simulations referring to those particular clinical areas are likely to be most successful. High fidelity simulation has demonstrated to improve PT students’ self-efficacy in acute care clinical practice [9–13]. Because self-efficacy is clinical case dependent, PT students should be able to practice with clinical cases derived from distinct clinical areas in order to develop a sense of self-efficacy in those clinical areas.

Another important finding of this study is the weak association between self-efficacy towards functioning as a physical therapist, and both general self-efficacy and work-related self-efficacy. This means that whilst some students might have strong general or work related self-efficacy beliefs, they might be uncertain about their functioning in clinical practice as a physical therapist. These findings are in line with Bandura’s suggestions that self-efficacy should be assessed in close relation to the context under study [34]. The implication of these findings is that in PT education self-efficacy beliefs should be assessed in relation to key criteria of functioning in one clinical area.

This study is not without its limitations. Firstly, all students were selected from only one educational institution (HAN University of Applied Sciences), and students had to volunteer to participate. This might have resulted in a selection bias. To further improve validity and reliability of the instrument, it is to be recommended that the scale be investigated in other institutional settings as well. A second drawback of the study is that the PSE is limited to three clinical areas. Students might be confronted with different cases not measured with the PSE. The PSE scores might thus not be a reliable measure for self-efficacy towards these other caseloads. However, the scale can easily be adapted for other clinical areas, for instance pain patients. Finally, it cannot be ruled out that answers to the PSE questions might be prone to effects of social desirability or acquiescence bias. Acquiescence bias is a tendency to agree with all the questions. Participating students were instructed to give honest answers to the questions, and that there is no good or bad answer. An indication that participants gave honest answers is the normal distributions of answers on all items. However, this does not rule out that some kind of bias might be at work.

Nonetheless, these findings have some important implications for PT education. Student self-efficacy beliefs should be assessed in relation to specific clinical areas. The PSE enables educators and researchers to measure improvement in the PT student’s self-efficacy towards physiotherapy functioning in relation to specific clinical areas. However, before the PSE is implemented further research is needed to improve the instrument. The PSE is a lengthy instrument (39 items) and some students complained about its length. Therefore, further research is needed to shorten the different scales. Based on further item analysis some items can be eliminated based on high inter-item correlations or conceptual overlap. However, additional research is needed to insure that the shortened scale’s ecological validity and integrity will remain intact. Based on these shortened scales, a confirmatory factor analysis is needed to confirm that the items in the improved scales accurately reflect the underlying constructs. In future research, the PSE instrument can be used both to help improve self-efficacy in the individual PT student, and to evaluate the effect of education on self-efficacy. The PSE can help the educator and the student to identify those areas of functioning into witch the students feels insecure and needs further practice. Research is needed into the merits of such a tailor made approach in PT education. In line with previous research [3], the PSE can also be used to evaluate the effect of different interventions on self-efficacy. Further research is needed to determine whether specific educational interventions are effective in increasing the students’ self-efficacy beliefs. Finally, as self-efficacy is considered an independent predictor for student performance in clinical settings [9], more research is needed to determine the predictive power of the PSE for future functioning in clinical practice.

## Conclusion

The PSE is a valid and reliable instrument to assess the student self-efficacy towards PT functioning in three distinct clinical areas. As such, it can be an important tool in education and education research for PT. In education, the PSE could be used to determine which skills the student feels less confident undertaking, and additional training/learning opportunities could be provided. In research, the PSE could be used to compare different educational methods in their effect on self-efficacy.

## Abbreviations

CI: Confidence Interval
COSMIN: Consensus-based Standards for the selection of health Measurement Instruments
D-GSES: Dutch General Self-efficacy Scale
EFA: Exploratory Factor Analysis
HAN: UAS Hogeschool Arnhem Nijmegen University of Applied Sciences
ICC: Intra class correlation
KMO: Kaiser-Maier-Olkin test
PsyCap: Psychological Capacity
PT: Physical Therapist
PTE: Physiotherapy self-efficacy
SD: Standard Deviation
SEM: Standard Error of Measurement
